# Evidence for Abasic Site Sugar Phosphate-Mediated Cytotoxicity in Alkylating Agent Treated *Saccharomyces cerevisiae*

**DOI:** 10.1371/journal.pone.0047945

**Published:** 2012-10-29

**Authors:** Michelle Heacock, Vladimir Poltoratsky, Rajendra Prasad, Samuel H. Wilson

**Affiliations:** Laboratory of Structural Biology, National Institute of Environmental Health Sciences, National Institutes of Health, Research Triangle Park, North Carolina, United States of America; Institute of Enzymology of the Hungarian Academy of Science, Hungary

## Abstract

To better understand alkylating agent-induced cytotoxicity and the base lesion DNA repair process in *Saccharomyces cerevisiae*, we replaced the *RAD27^FEN1^* open reading frame (ORF) with the ORF of the bifunctional human repair enzyme DNA polymerase (Pol) β. The aim was to probe the effect of removal of the incised abasic site 5′-sugar phosphate group (i.e., 5′-deoxyribose phosphate or 5′-dRP) in protection against methyl methanesulfonate (MMS)-induced cytotoxicity. In *S. cerevisiae*, Rad27^Fen1^ was suggested to protect against MMS-induced cytotoxicity by excising multinucleotide flaps generated during repair. However, we proposed that the repair intermediate with a blocked 5′-end, i.e., 5′-dRP group, is the actual cytotoxic lesion. In providing a 5′-dRP group removal function mediated by dRP lyase activity of Pol β, the effects of the 5′-dRP group were separated from those of the multinucleotide flap itself. Human Pol β was expressed in *S. cerevisiae*, and this partially rescued the MMS hypersensitivity observed with *rad27^fen1^*-null cells. To explore this rescue effect, altered forms of Pol β with site-directed eliminations of either the 5′-dRP lyase or polymerase activity were expressed in *rad27^fen1^*-null cells. The 5′-dRP lyase, but not the polymerase activity, conferred the resistance to MMS. These results suggest that after MMS exposure, the 5′-dRP group in the repair intermediate is cytotoxic and that Rad27^Fen1^ protection against MMS in wild-type cells is due to elimination of the 5′-dRP group.

## Introduction

Endogenous and exogenous agents constantly damage the genome in all organisms. This damage must be repaired for the cell to avoid genomic instability or cell death. To counteract DNA damage, organisms employ several distinct DNA repair pathways that are designed to correct specific types of DNA damage. Base excision repair (BER) is one such pathway repairing smaller base lesions, single-strand breaks in DNA, and abasic sites resulting from spontaneous hydrolysis at the glycosidic bond. In the case of base lesions, repair is usually initiated by a lesion-specific DNA glycosylase. Glycosylases are classified as either monofunctional or bifunctional. Monofunctional glycosylases remove a damaged base leaving the abasic site in double-stranded DNA, while bifunctional glycosylases perform base removal and have the additional function of lyase-mediated incision of the phosphodiester DNA backbone leaving a single-strand break [1].

The BER sub-pathway that is utilized for repair of methylated DNA bases, arising after cell treatment with the DNA methylating agent methyl methanesulfonate (MMS), involves base removal by the monofunctional DNA glycosylase termed MAG [1], [2]. This is followed by DNA strand incision by an AP endonuclease, creating a 1-nt gap with 5′-deoxyribose phosphate (5′-dRP) and 3′-OH groups at the margins [1], [3]. This 5′-dRP group provides an important role in mammalian BER where it is recognized by the BER factor PARP-1 [4] that promotes recruitment of other repair factors to the BER intermediate [5]. In mammalian BER, the 5′-dRP group is removed by Pol β and this enzyme also inserts a single nucleotide to fill the gap [6]. Thus, Pol β has two functions in mammalian BER, and the respective enzymatic activities are carried in dedicated domains: the 8-kDa dRP lyase domain and 31-kDa DNA polymerase domain [7]. It was shown previously that these respective activities could be eliminated by site-directed alterations of amino acids without substantially altering the other activity. Thus, strategic Lys to Ala substitions in the dRP lyase active site (i.e., Lys35, Lys68 and Lys72 to Ala) eliminate the dRP lyase activity with little effect on the polymerase activity, and similarly, an Asp to Ala substitution in the polymerase active site (i.e., D256A) eliminates the polymerase activity without changing the dRP lyase activity [8].

In many organisms studied to date, the BER process occurs by two sub-pathways differentiated by repair patch size and the factors involved. These sub-pathways are termed single-nucleotide (SN) or short patch BER (insertion of only one nucleotide into the repair patch) and long patch (LP) BER (insertion of 2 or more nucleotides into the repair patch) [9], [10]. Higher eukaryotes often appear to preferentially use SN BER to remove the 5′-dRP group and fill the 1-nt gap, except in cases where a modified 5′-dRP group is refractory to the dRP lyase activity. In a case, where the dRP lyase activity of Pol β is unable to remove a dRP group, LP BER is evoked [6]. LP BER is achieved either through strand-displacement DNA synthesis by replicative and repair DNA polymerases along with Fen1 flap excision or by coordinated action of Pol β and Fen1 [11], in both cases creating a substrate for DNA ligation. While higher eukaryotes are able to perform both sub-pathways of BER, *S. cerevisiae* appears to rely primarily on LP BER and on other repair pathways to repair smaller base lesions [12].

A model for repair of methylated bases in *S. cerevisiae* begins with the same upstream BER events as summarized above for mammalian cells. The DNA glycosylase responsible for removal of methylated bases, methylpurine DNA gylcosylase, creates an abasic site intermediate [2]. Strand incision by AP endonuclease (APN1) creates the 5′-dRP group at the margin of a single-nucleotide gap. Next, the gap and strand break are processed by LP BER or mobilized to participate in recombinational repair.

The deletion of *RAD27^FEN1^* in *S. cerevisiae* is known to render these cells hypersensitive to MMS [13], [14], [15]. However, it has not been clear whether this is due to: 1) the alkylated base lesions themselves, 2) the persistence of repair intermediates such as the abasic site or the 5′-dRP group in the repair intermediate, 3) the accumulation of a toxic multinucleotide flap [16], [17]. One approach toward clarifying the picture is to express an enzyme dedicated to removing the 5′-dRP group in the repair intermediate in a *rad27^fen1^*-null background. Thus, Pol β expression would be expected to provide for 5′-dRP group removal. In the work described here we found that deletion of *RAD27^FEN1^* resulted in hypersensitivity to MMS, as expected [13], [14], [15], and human Pol β expression partially rescued the MMS hypersensitivity imposed by *RAD27^FEN1^* deletion. It was the 5′-dRP lyase function of Pol β, not the DNA polymerase function, that conferred resistance to MMS. The results are discussed in the context of toxicity of the 5′-dRP blocking group during repair in *S. cerevisiae*.

## Materials and Methods

### Materials

Synthetic oligodeoxyribonucleotides were from Oligos Etc., Inc. (Wilsonville, OR) and The Midland Certified Reagent Co. (Midland, TX). [α-^32^P]dCTP and [α-^32^P] 3′-deoxyadenosine (Cordycepin) (3000 Ci/mmol) were from GE Healthcare (Piscataway, NJ). [γ-^32^P]ATP (7000 Ci/mmol) was from Biomedicals (Irvine, CA). Optikinase and terminal deoxynucleotidyl transferase were from USB Corp. (Cleveland, OH) and Fermentas Inc. (Hanover, MD). Recombinant human Pol β was overexpressed and purified as described previously [18]. Human uracil DNA glycosylase with 84 amino acids deleted from the amino-terminus and DNA ligase I were purified as described previously [19], [20]. Protease inhibitor tablets were from Roche (Indianapolis, IN). MMS and camptothecin (CPT) were from Sigma-Alrich (St. Louis, MO). Phusion polymerase was from New England Biolabs (Ipswich, MA). Goat-anti-mouse secondary antibody conjugated to horseradish peroxidase (HRP) was purchased from Bio-Rad (Hercules, CA). Super Signal West Pico Western Blot Substrate (ECL) and Restore Western Blot Stripping Buffer were from Pierce Biotechnology Inc. (Rockford, IL). Mouse anti-GAPDH antibody was obtained from Alpha Diagnostics (San Antonio, TX).

### Strain construction

All strains (Table 1) were derived from the yeast strains ALE1000 and ALE1001 (*MATα-HML/Rδ leu2-3112 ade5-1 his 7-2 ura3δ trip1-289* BAR+ [(chr II) *lys2::Alu-DIR-LEU2-lys2δ5*′]). The *rad27*::CORE strain (*rad27*Δ) was constructed by inserting the delitto perfetto *CORE-I-SCEI* cassette containing URA3 and hygromycin (HYGRO) markers [21]) into the *RAD27* locus. The *CORE-I-SCEI* was amplified using the forward primer, Rad27coref, 5′ ATATACATCGATGAAAAGCGTTGACAGCATACATTGGAAAGAAATAGGAAACGGAACCGGAAGAAAAAATTAGGGATAACAGGGTAATCCGCGCGTTGGCCGATTCAT 3′ and the reverse primer, Rad27corer, 5′ GTATACAAATATCTATGTTACATATATGCCAAGGTGAAGGACCAAAAGAAGAAAGTGGAAAAAGAACCCCCTCATTCGTACGCTGCAGGTCGAC 3′. Hygromycin-resistant transformants were selected following the methods described by Storici and Resnick [21]. These transformants were genotyped using the Rad27f, 5′ CGTAACATCGCGCAAATGAAGGTT 3′ and the Rad27r, 5′ TATTAGAAATTCCACCGGCACCTG 3′ primers. The *rad27*::*POLβ* strain was constructed using homologous recombination of a PCR product generated using the Polbrad27f, 5′ TATATACATCGATGAAAAGCGTTGACAGCATACATTGGAAAGAAATAGGAAACGGACACCGGAAGAAAAAATATGTCTAAACGGAAGGCGCCGCAG 3′ and the Polbrad27r, 5′GTATACAAATATCTATGTTACATATATGCCAAGGTGAAGGACCAAAAGAAGAAAGTGGAAAAAGAACCCCCTCATTCGCTCCGGTCCTTGGGTTC 3′ primers. The PCR product was amplified using either wild-type Pol β, polymerase-deficient Pol β (*polβ-D256A*) or lyase-deficient Pol β (*polβ K35A/K68A/K72A* and termed as *polβ-3K*) cDNA as a substrate [8]. PCR reactions (50 µl total volume) consisted of 10 ng of DNA substrate, 0.2 µM of each primer, 50 µM of dNTPs, 1× reaction buffer (50 mM KCl, 20 mM Tris-HCl, pH 8.4, 1.5 mM MgCl_2_, 0.05% Tween-20), 1 U of Phusion polymerase. The two cycle PCR program 98°C 2 min, 5 cycles of 98°C 10 sec, 55°C 30 sec, 72°C 1 min was followed by 20 cycles of 98°C 10 sec, 68°C 30 sec, 72°C 1 min with a final extension of 7 min. PCR products were purified using Qiagen columns, eluted in 50 µl water and 1 µg was used in each transformation using the LiCl method. Transformation of PCR products were made into the *rad27*::CORE strain. Positive transformants were obtained by their ability to grow on 5-fluoroorotic acid followed by PCR genotyping using primers upstream and downstream of the *RAD27* locus with the forward primer, Rad27f, 5′ CGTAACATCGCGCAAATGAAGGTT 3′ and the reverse primer, Rad27r, 5′ TATTAGAAATTCCACCGGCACCTG 3′. All strains were sequenced to verify the respective *POLβ* mutation.

**Table 1:** *S. cerevisiae* strains used in this study.

Wild type strain background is: MATα-HML/Rδ *leu2-3112 ade5-1 his 7-2 ura3δ trp1-289* BAR+ [(chr II) *lys2::Alu-DIR-LEU2-lys2δ5′*] [16]			
Name	Strain	Relevant Genotype	Source
*rad27*Δ	VP101	*rad27*::CORE	This study
*rad27*::*POLβ*	VP102	*rad27*::*POLβ-WT*	This study
*rad27*::*polβ-D256A*	MH101	*rad27*::*polβ-D256A*	This study
*rad27*::*polβ-3K*	MH102	*rad27*::*polβ-3K*	This study

Wild type strain background for *mre11* and *rad50* is MATa *his3Δ0 leu2Δ0 met15Δ0 ura3Δ0* (Research Genetics, Inc., Huntsville, AL)			
Name	Strain	Relevant Genotype	Source
*mre11Δ*	*mre11::KanMX*	*mre11::KanMX*	Research Genetics, Inc., Huntsville, AL
*rad50Δ*	*rad50::KanMX*	*rad50::KanMX*	Research Genetics, Inc., Huntsville, AL

### Immunoblotting

Total protein extracts were prepared using 25 ml of mid-log phase cells. Cells were collected by centrifugation and resuspended in 500 µl ice-cold lysis buffer (25 mM Tris-HCl, pH 7.5, 1 mM EDTA, 100 mM NaCl, and 10 mM β-mercaptoethanol, supplemented with a protease inhibitor tablet. Cells were disrupted using the glass bead method, adding an equal volume of 500 µl glass beads and vortexing using 1-min increments, placing samples on ice between vortexing cycles. Protein concentration was determined using the Bradford method. Protein extract (50 µg) mixed with SDS-PAGE loading buffer was used per lane. Proteins were separated in a 4–12% Bis-Tris polyacrylamide gel electrophoresis. Proteins were transferred onto a nitrocellulose membrane using wet transfer. After blocking with 5% milk in Tris-buffered saline containing Tween 20, the membrane was incubated for at least 2 h with a mouse monoclonal antibody to Pol β (18S). Goat anti-mouse conjugated to HRP was used as a secondary antibody (1∶5000 dilution), and the HRP activity was detected by enhanced chemiluminescence using SuperSignal West Pico Chemiluminescent substrate. For loading controls, blots were stripped using Restore Western Blot Stripping Buffer, probed with mouse anti-GAPDH (1∶10000 dilution) and visualized as above.

### Preparation of 5′- and 3′-end labeled substrates for dRP lyase assays

Preparation of the 5′- or 3*′*-end ^32^P-labeled DNA substrate for dRP lyase assay was as follows. Dephosphorylated 19-mer oligodeoxyribonucleotide (5′-UGTACGGATCCCCGGGTAC-3′) containing a uracil residue at the 5′-end or 3′-end was phosphorylated with Optikinase and [γ-^32^P]ATP or 3′-end with terminal deoxynucleotidyl transferase and [α-^32^P] Cordycepin, respectively. A 34-mer (5′-GTACCCGGGGATCCGTACGGCGCATCAGCTGCAG-3′) template was then annealed with 15-mer (5′-CTGCAGCTGATGCGC-3′) and 19-mer ^32^P-labeled oligonucleotides by heating the solution at 90°C for 3 min and allowing the solution to slowly cool to 25°C. Unincorporated [γ-^32^P]ATP or [α-^32^P] Cordycepin was removed by using a MicroSpin™ G-25 column (GE HealthCare) using the manufacturer's suggested protocol.

### dRP lyase assay

dRP lyase activity was measured essentially as described previously [22]. Briefly, the reaction mixture (10 µl) contained 50 mM HEPES, pH 7.5, 20 mM KCl, 2 mM dithiothreitol, 0.5 mM EDTA, 5 mM MgCl_2_, and 100 nM 3′-end ^32^P-labeled uracil-containing DNA substrate. The reaction was initiated by adding 4 µg yeast extract or Pol β (10 nM). In the case of purified Pol β, the reaction mixture contained DNA substrate pre-treated with UDG. The incubation was for 15 min at 35°C. After the incubation, the reaction products were stabilized by the addition of freshly prepared 20 mM NaBH_4_, then transferred to 0–1°C (on ice) and incubation continued for 30 min on ice. After further incubation at 75°C for 2 min, the reaction products were separated by electrophoresis in a 16% polyacrylamide gel containing 8 M urea in 89 mM Tris-HCl, 89 mM boric acid, and 2 mM EDTA, pH 8.8. Imaging and data analysis were performed by PhosphorImager™ and ImageQuant™ software. In an alternate dRP lyase reaction, the dRP lyase reaction was performed as above, except 5′-end labeled DNA substrate was used.

### *In vitro* BER assay

To perform the uracil-initiated SN BER with yeast extracts, a 35-base pair oligonucleotide duplex DNA containing uracil at position 15 was used. The BER reaction mixture (15 µl final volume) that contained 50 mM HEPES, pH 7.5, 0.5 mM EDTA, 2 mM dithiothreitol, 20 mM KCl, 4 mM ATP, 5 mM phosphocreatine, 100 µg/ml phosphocreatine kinase, 0.5 mM NAD^+^, 5 mM MgCl_2_, 200 nM DNA ligase I, 200 nM uracil-containing DNA, and 2.3 µM [α-^32^P]dCTP (specific activity, 1×10^6^ dpm/pmol) was assembled on ice. The repair reaction was initiated by the addition of 6 µg yeast extract and incubation at 35°C. Aliquots (4.5 µl) were withdrawn at the indicated time intervals. Reactions were terminated by the addition of an equal volume (4.5 µl) of DNA gel-loading buffer. After incubation at 75°C for 2 min, the reaction products were processed as above.

### Primer extension assay

The primer extension assay system involved a 36-mer template strand annealed with a 17-mer 5′-^32^P-labeled primer; the template strand ahead of the primer was single-stranded. The reaction mixture (15 µl final volume) containing 50 mM HEPES, pH 7.5, 0.5 mM EDTA, 2 mM dithiothreitol, 20 mM KCl, 10 mM MgCl_2_, 100 µM dNTPs, and 200 nM DNA was assembled on ice. The repair reaction was initiated by the addition of 9 µg yeast extract and incubation at 35°C. Aliquots (4.5 µl) were withdrawn at 15, 30 and 60 min. Reactions were terminated by the addition of an equal volume (4.5 µl) of DNA gel-loading buffer. After incubation at 75°C for 2 min, the reaction products were processed as above.

### MMS sensitivity spot dilution assay

Each strain was freshly streaked onto yeast peptone dextrose adenine (YPDA) plates from frozen stocks and used to start 5 ml liquid YPDA cultures that were grown overnight. A fresh culture of the same volume was prepared by inoculation with the overnight culture and allowed to grow at least 4 h (until reaching an OD_600_ of ∼0.5, where the OD_600_ was normalized for each strain using the lowest OD_600_). Ten-fold dilutions (shown on plates) were set-up for each strain and cultures (5 µl of each dilution) were spotted onto YPDA plates containing either no MMS (mock), 0.5, or 1.0 mM MMS. Plates were incubated at 30°C and photographed 2–3 days after growth. All experiments were done in triplicate.

### CPT sensitivity spot dilution assay

A 10 mM stock solution of CPT was prepared by dissolving it in dimethyl sulfoxide (DMSO) and aliquots were stored at −20°C until use. Dilutions of each strain and culture were performed as described above, except cultures were spotted onto plates containing DMSO alone (mock), 25 µM or 50 µM CPT. Results with 25 µM CPT are shown. All experiments were done in triplicate.

### Gamma irradiation spot dilution assay

Each strain was diluted and spotted as described for MMS sensitivity spot assays. Immediately upon drying, plates containing the spotted dilutions were subjected to mock, 200 or 600 Gray of γ-irradiation using a ^137^Cs irradiator. Results with 200 Gray of γ-irradiation are shown. Photographs were taken 3 days after plating.

## Results

### Human Pol β is expressed in active form in *S. cerevisiae*

**Figure 1:**
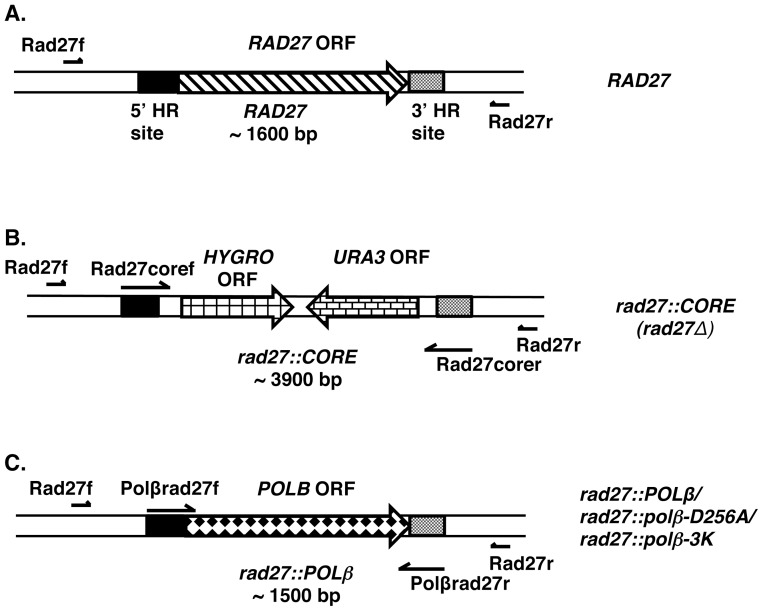
Strategy for introduction of a human DNA *POLβ* open reading frame into the *S. cerevisiae* genome. (A) Schematic representation of the RAD27 gene (∼1600 bp) of *S. cerevisiae* is shown. The positions of the RAD27 5′ and 3′ homologous recombination integration sites (5′ and 3′ HR sites, respectively), relative to the 5′ and 3′ regions of the Rad27 gene, are indicated. The positions of the forward primer (Rad27f) and the reverse primer (Rad27r) used for genotyping are also illustrated. (B) First, the *CORE-I-SCEI* was amplified using the Rad27coref and Rad27corer primers. The *rad27*::*CORE* strain was constructed by homologous recombination of a PCR product containing the *CORE-I-SCEI* cassette containing URA3 and hygromycin (HYGRO) markers flanked by up-and-downstream RAD27 sequence. Hygromycin-resistant transformants were genotyped using the Rad27f and Rad27r primers. Correct integration of the CORE cassette resulted in a PCR product of ∼3900 bp. Failure to integrate resulted in wild-type product of ∼1600 bp. (C) The strains of *rad27*::*POLβ*, *rad27*::*polβ -D256A, and rad27*::*polβ -3K* were generated by homologous recombination of a PCR product of POLB open reading frame using primers Polbrad27f and Polbrad27r. Replacement of the CORE cassette by human POLβ open reading frame was confirmed using primers Rad27f and Rad27r that resulted in a PCR product of ∼1500 bp.

We developed a system for study of 5′-dRP group toxicity in *S. cerevisiae* by deleting the *RAD27* gene and inserting the *POLβ* ORF in its place, under control of the *RAD27* promoter (Figure 1). Thus, we prepared a strain with full-length wild-type human *POLβ* at the *RAD27* locus and two strains with point mutations eliminating the respective activities in each of the *POLβ* domains, a polymerase-deficient mutant (*polβ-D256A*) [23] and a lyase-deficient mutant (*polβ-3K*) [8]. The strains are summarized in Table I, and features of the strains are summarized in Figure 1.

**Figure 2:**
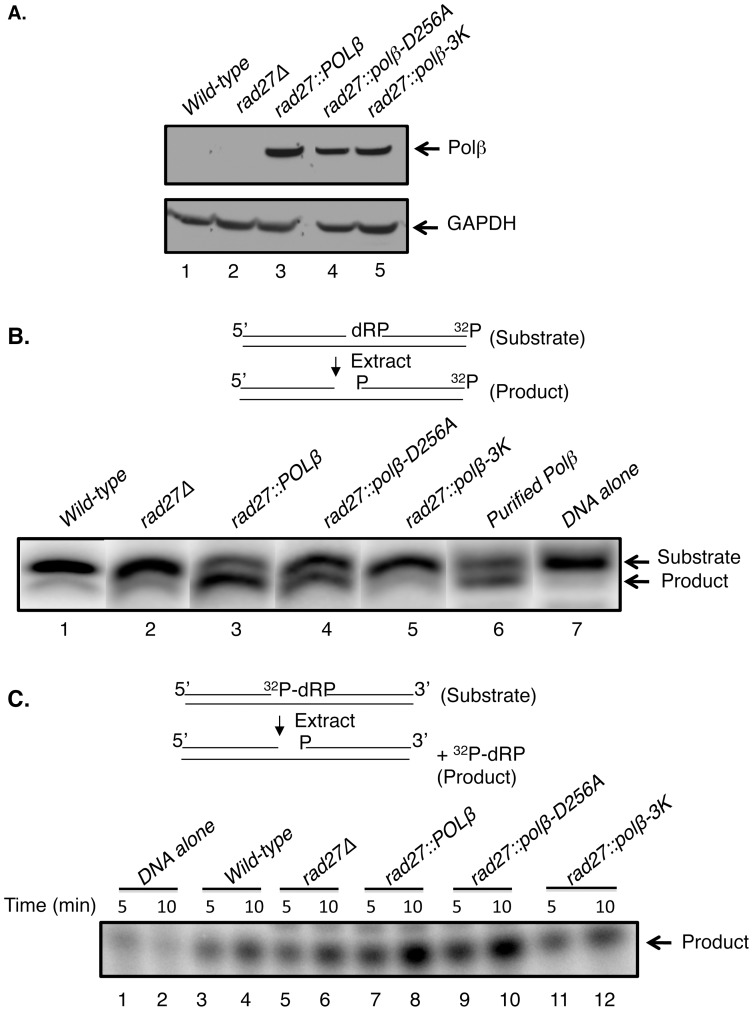
Expression of human *POLβ* into *S. cerevisiae rad27* mutants. (A) Immunoblot illustrating human Pol β expression in *S. cerevisiae*. Total extract protein was isolated (see Materials and Methods) from the indicated five strains. The indicated extracts (50 µl each) from *wild-type* (lane 1), *rad27*Δ (lane 2), *rad27::POLβ* (lane 3), *rad27::polβ-D256A* (lane 4), or *rad27::polβ-3K* (lane 5), respectively, were separated by SDS-PAGE, transferred to a nitrocellulose membrane, and probed either with an antibody against pol β (upper panel) or with an antibody against GAPDH (bottom panel). Bands corresponding to Pol β (39 kDa) were observed. GAPDH was used as a loading control. (B and C) 5′-dRP lyase assays. Schematic representations of the dRP lyase substrates (3′- or 5′-end ^32^P-labeled) and the expected ^32^P-labeled products formed as a result of lyase activity of the extract are illustrated. (B) DNA substrate (100 nM) was incubated with extracts from *wild-type* (lane 1), *rad27*Δ (lane 2), *rad27::POLβ* (lane 3), *rad27::polβ-D256A* (lane 4), and *rad27::polβ-3K* (lane 5), with *purified* Pol β (lane 6), or with DNA alone (lane 7). After a 15-min incubation at 35°C, the DNA products were stabilized by NaBH_4_ and analyzed as described under Material and Methods. The positions of the substrate and product are indicated. (C) 5′-end ^32^P-labeled DNA substrate (100 nM) was incubated either with DNA alone (lanes 1–2) or with extracts from *wild-type* (lanes 3–4), *rad27*Δ (lanes 5–6), *rad27::POLβ* (lanes 7–8), *rad27::polβ-D256A* (lanes 9–10), and *rad27::polβ-3K* (lane11–12), respectively. After 5 and 10 min incubations at 35°C, the DNA products were stabilized and processed as in (B). The position of the product is indicated.

First, to verify Pol β expression in the strains, we performed immunoblotting analysis of whole cell extracts using an anti-Pol β antibody (Figure 2A). Pol β was present in all three strains where the *POLβ* ORF had been introduced and the levels appeared to be similar (Figure 2A, lanes 3–5); in the cases of the wild-type and *rad27-*deletion strains (*rad27Δ*, Figure 2A, lanes 1 and 2), no signal was observed corresponding to Pol β, as expected.

We also used these cell extracts to verify the functional status of the dRP lyase activity of the different forms of expressed Pol β. Two assays designed to measure removal of the 5′-dRP group from oligonucleotide substrates were used (Figure 2B and C). The dRP lyase activity was functional in the *rad27*::*POLβ* and *rad27::polβ-D256A* strains (Figure 2B, lanes 3 and 4), but not in the wild-type, *rad27Δ*, or *rad27::polβ-3K* strain (Figure 2B, lanes 1, 2 and 5), as expected. These results were confirmed by making use an alternate assay for dRP lyase activity (see Figure 2C). In this alternate assay, the substrate contained a ^32^P-labeled dRP flap in a single-nucleotide gap, as shown at the top of Figure 2C. The results obtained with this assay were similar to those obtained with the other assay (Figure 2B). Note that the wild-type and *rad27Δ* strains exhibited relatively weak dRP lyase activity (Figure 2B and C).

### BER status of the strains

To evaluate the status of BER in strains studied here, we characterized the *in vitro* BER capacity of cell extracts (Figure S1). A BER substrate was incubated with extracts as described in Material and Methods. Initial experiments were conducted with the wild-type and *rad27::POLβ* strain extracts. Extract from the wild-type strain had minimal BER activity (Figure S1, lanes 1–3); in the *rad27::POLβ* extract, strong BER gap-filling synthesis was observed, as indicated by the incorporation of radiolabeled dCMP (Figure S1, lanes 4–6). Yet, the complete repair product was not observed. Addition of purified DNA ligase I, however, resulted in strong synthesis of the ligated BER product (Figure S1, lanes 7–9). These results indicated only weak BER activity in the wild-type extract, as previously observed [24], and also suggested a deficiency in DNA ligase activity in the extracts. Therefore, in further assays purified DNA ligase I was included in the reaction mixtures.

**Figure 3:**
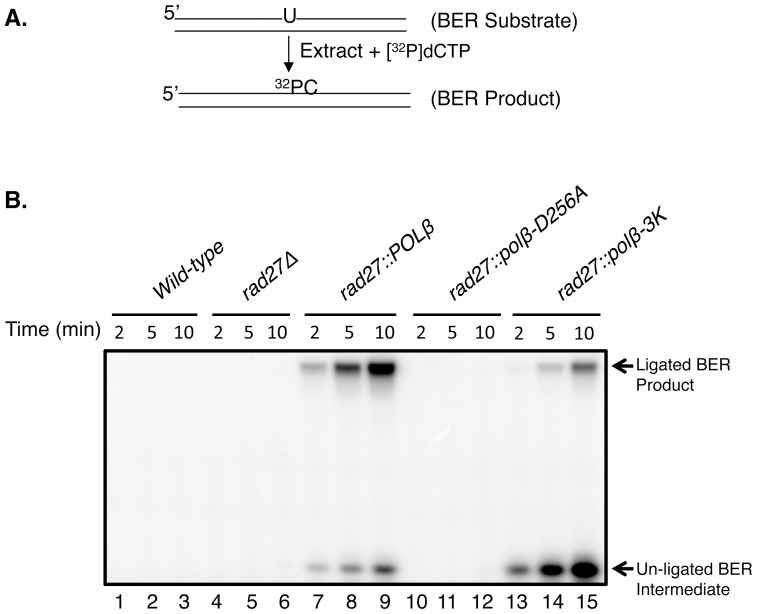
*In vitro* BER capacity of *S. cerevisiae* extracts. (A) Schematic representations of the substrate and the reaction scheme are shown. (B) Repair reactions (15 µl each) were incubated either with extracts from *wild-type* (lanes 1–3), *rad27*Δ (lanes 4–6), *rad27::POLβ* (lanes 7–9), *rad27::polβ-D256A* (lanes 10–12), or *rad27::polβ-3K* (lane13–15), respectively. Note that all the reaction mixtures were supplemented with human DNA ligase I (200 nM). Aliquots (4.5 µl each) were withdrawn at 2, 5 and 10 min. The repair reaction was terminated by addition of an equal volume of DNA gel-loading buffer. After incubation at 75°C for 2 min, the reaction products were separated by electrophoresis in a 16% polyacrylamide gel containing 8 M urea. A Typhoon PhosphorImager was used for gel scanning and imaging. The positions of ligated BER product and un-ligated BER intermediate are indicated.

**Figure 4:**
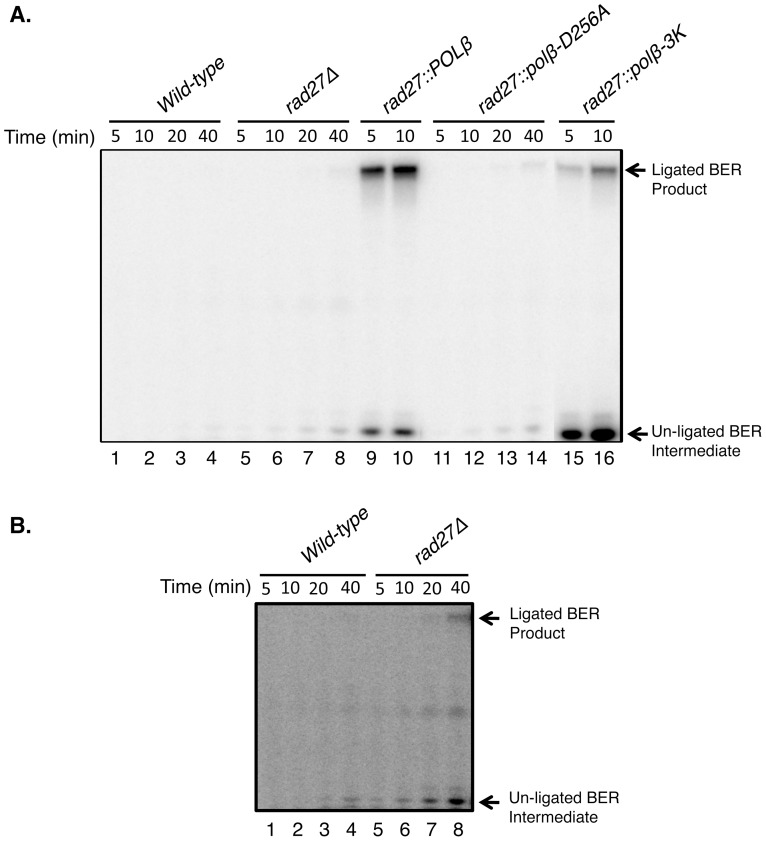
*In vitro* BER capacity of *S. cerevisiae* extracts. (A) Repair reactions were incubated either with extracts from *wild-type* (lanes 1–4), *rad27*Δ (lanes 5–8), *rad27::POLβ* (lanes 9–10), *rad27::polβ-D256A* (lanes 11–14), or *rad27::polβ-3K* (lanes15–16), respectively. Note that all the reaction mixtures were supplemented with human DNA ligase I (200 nM) as in Figure 4. Reaction mixtures were incubated at 35°C and samples were withdrawn at the indicated periods. After incubation, the reaction products were processed as in Figure 3. The positions of ligated BER product and un-ligated BER intermediate are indicated. (B) Lanes 1 to 8 from the panel (A) were exposed for a longer time to observe BER products in the *wild-type* (lanes 1–4) and *rad27*Δ (lanes 5–8) strains.

To evaluate the *in vitro* BER activity of the strains with mutant forms of Pol β, BER assays were conducted using extracts from the complete panel of strains (Figures 3 and 4). Under the reaction condition, wild-type, *rad27*Δ and *rad27::polβ-256A* extracts showed negligible BER activity (Figure 3, lanes 1–6 and 10–12). In contrast, the *rad27::POLβ* extract was active for BER. With the *rad27::polβ-3K* extract, formation of some ligated product was detected, but the amount was less than in the *rad27::POLβ* extract. These results indicated that endogenous 5′-dRP lyase activity was present, but was insufficient to complement the Pol β 5′-dRP lyase deficiency of the *rad27::polβ-3K* strain (Figure 3, lanes 13–15); nevertheless, the production of some full-length BER product in these reactions indicated that the 5′-dRP group had been removed from at least a portion of the gap-filled BER intermediates.

**Figure 5:**
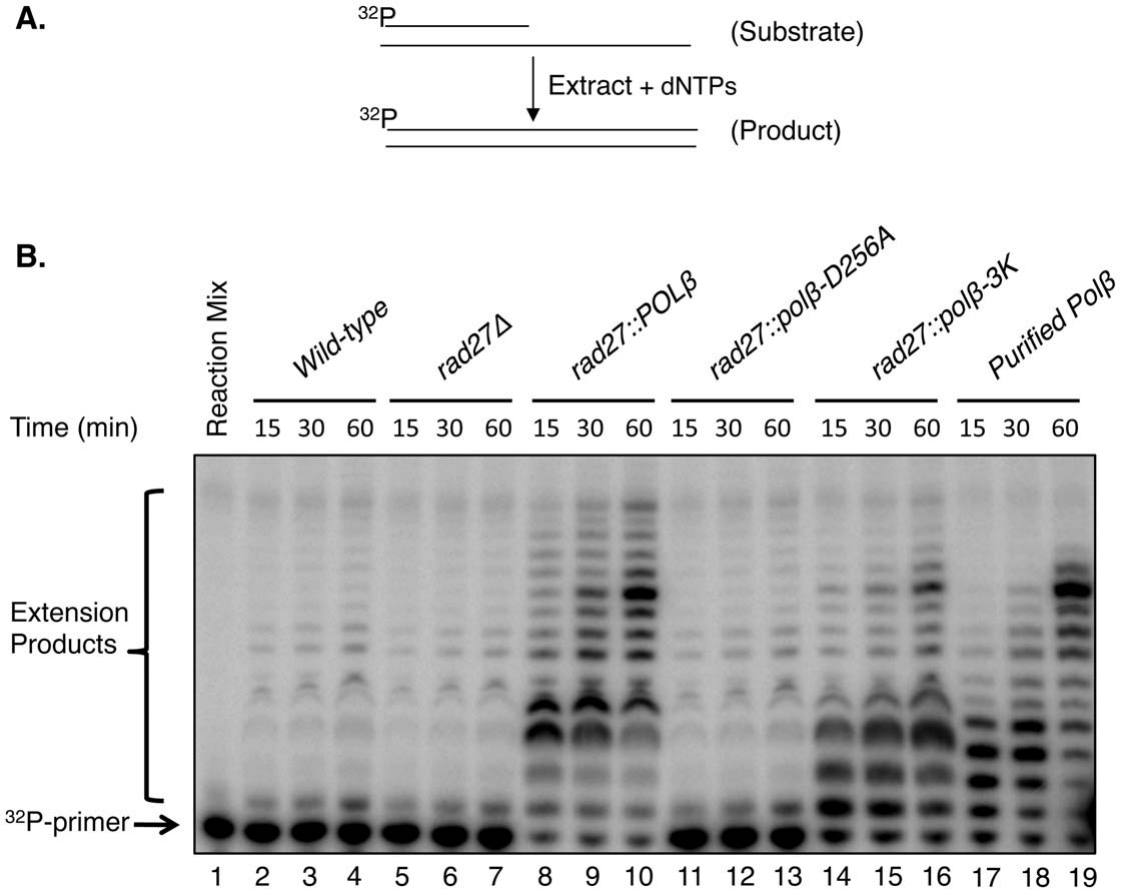
*In vitro* primer extension assay by *S. cerevisiae* extracts. (A) Schematic representations of the substrate and the reaction scheme are shown. (B) The primer extension reaction mixture was incubated either with reaction mixture alone (lane 1), or with extracts from *wild-type* (lanes 2–4), *rad27*Δ (lanes 5–7), *rad27::POLβ* (lanes 8–10), *rad27::polβ-D256A* (lanes 11–13), *rad27::polβ-3K* (lanes14–16), and purified pol β (lanes 17–19), respectively. Reaction mixtures were incubated at 35°C and samples were withdrawn at the indicated periods. After incubation, the reaction products were processed as in Figure 3. The positions of ^32^P-primer and the extension products are indicated.

The fact that negligible BER activity was observed with the wild-type and *rad27*Δ extracts raised a concern that the extracts were somehow denatured or defective for DNA synthesis. Therefore, to evaluate this, the BER reaction mixtures were incubated for extended periods as compared to the BER reactions in Figure 3. Under these reaction conditions, formation of some un-ligated BER intermediates was observed, yet the BER capacity of these extracts was minimal (Figure 4 A and B). Next, we conducted DNA polymerase primer extension experiments with the extracts. These experiments revealed that the extracts were capable of DNA polymerase activity in a primer extension assay with an open template strand ahead of the primer (Figure 5). Thus, the extracts were functional for DNA synthesis activity, and the fact that negligible or weak BER activity was observed with wild-type and other extracts (Figure 3, lanes 1–6 and 10–12) appeared to be due to the substrate specificity of the endogenous DNA polymerases.

### Human *POLβ* partially rescues MMS sensitivity observed in the absence of *RAD27*

**Figure 6:**
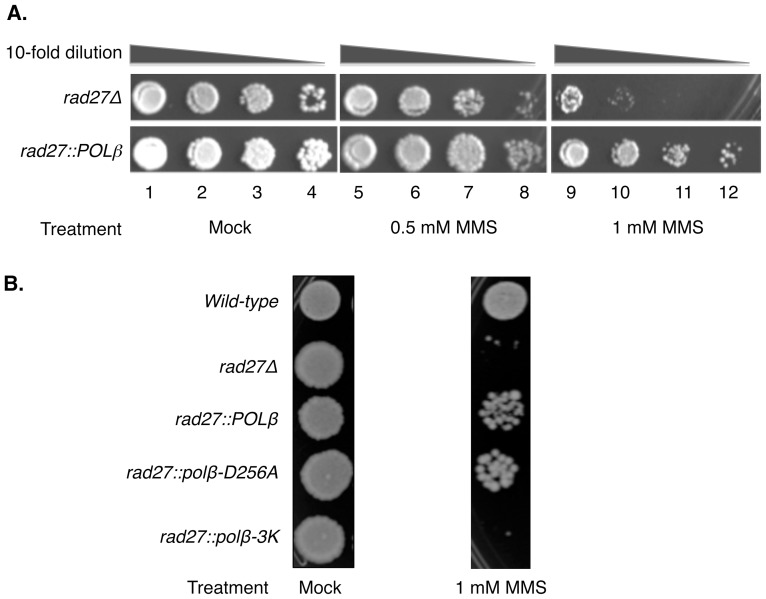
Human *POLβ* rescues MMS sensitivity in *S. cerevisiae*, null for *rad27*. Experiments were conducted as described in Materials and Methods. Briefly, ten-fold dilutions (as shown) were set-up for each strain and culture (5 µl of each dilution) were spotted onto freshly prepared YPDA plates containing the indicated concentration of MMS. Photographs were taken on day 3 or 4 after plating. (A) Mock treatment, lanes 1–4; 0.5 mM MMS treatment, lanes 5–8; 1 mM MMS treatment, lanes 9–12. (B) Rescue of MMS sensitivity by human Pol β dRP lyase. Sensitivity to MMS was scored by spotting cultures of various strains on plates either without (mock) or with 1 mM of MMS. Resistance to MMS was observed in *rad27* null strains that contain *POLβ* or *polβ-D256A*. Lack of cell growth was observed only in the *rad27* null strains (*rad27*Δ) or the strain containing mutations in the dRP lyase domain (*rad27*::*polβ-3K*). Strains are indicated on the left.

Deletion of *RAD27* causes a hypersensitivity phenotype following exposure of the DNA methylating agent MMS [13], [14], [15]. To test the ability of Pol β to rescue this MMS hypersensitivity, we used spot dilution assays on YPDA plates containing two concentrations of MMS (0.5 and 1 mM). Both *rad27Δ* and *rad27*::*POLβ* strains were able to grow on control (mock) plates that did not contain MMS (Figure 6A). On plates containing MMS, cell growth was diminished with the strain deleted for *RAD27*, indicating hypersensitivity. The MMS sensitivity was less when the *POLβ* ORF was expressed in the *rad27*-deleted strain (Figure 6A).

### The 5′-dRP lyase of Pol β is required for rescue of MMS sensitivity

Pol β is a bifunctional BER enzyme and catalyzes two distinct reactions, namely dRP lyase and DNA polymerase. We utilized the strains described above to determine the contribution of each enzymatic function in protection against MMS (Figure 6B). All strains grew well on control (mock) plates. Sensitivity to 1 mM MMS was pronounced in the lyase-deficient strains, *rad27*Δ and *rad27::polβ-3K*. However, sensitivity was less with the *POLβ* wild-type (*rad27::POLβ*) strain and the polymerase deficient-lyase proficient (*rad27::polβ-D256A*) strain (Figure 6B). The results demonstrated that the Pol β-dependent protection against MMS cytotoxicity in the absence of Rad27 did not require the polymerase activity, but instead the dRP lyase function was critical. These results also indicated that the Pol β DNA polymerase activity itself did not promote cytotoxicity.

**Figure 7:**
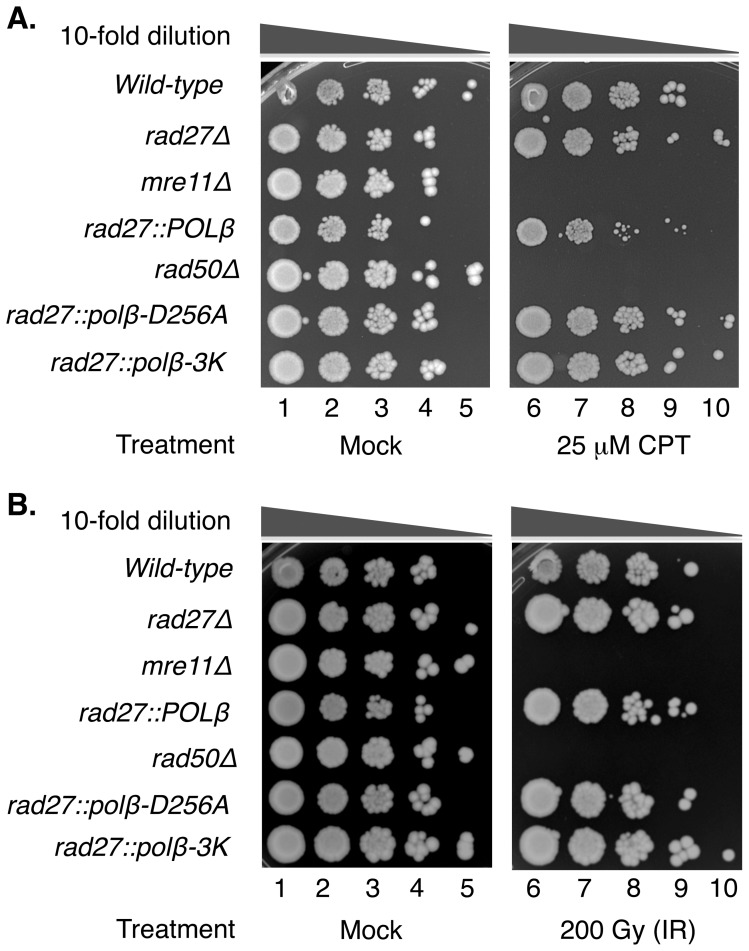
Rescue of CPT and ionizing radiation sensitivity by human Pol β dRP lyase. (A) Ten-fold dilutions of each indicated strain were spotted onto plates containing either DMSO alone (mock, lanes 1–5) or 25 µM CPT (lanes 6–10). *POLβ* strains were not sensitive to CPT, an agent that produced 3′ blocking groups. *mre11*Δ and *rad50*Δ deleted strains were used as positive controls for CPT-sensitivity. (B) *POLβ* strains do not display sensitivity to ionizing radiation (IR), an agent that produces predominately 3′ blocking groups. As in (A), *mre11*Δ and *rad50*Δ deleted strains were used as positive controls for IR sensitivity. Indicated strains were plated and treated as described in Material and Methods, and were mock-treated (lanes 1–5) or subjected to 200 Gray (Gy) of IR, lanes 6–10.

The failure of the lyase-deficient strain to rescue MMS sensitivity may be caused by accumulation of unligatable nicks with the 5′-dRP blocking group. A prediction of this interpretation is that strains deficient in 5′-dRP lyase function would not be selectively affected by damaging agents that fail to result the 5′-dRP blocking group. Therefore, we next examined the sensitivity of the strains to CPT. Since CPT acts by trapping the topoisomerase enzyme at the 3′-margin of a single-strand break, a 5′-dRP blocking group is not involved in the repair. Multiple strains were grown on plates containing 25 µM CPT (Figure 7A). Sensitivity was not observed for any of the strains plated on mock plates (DMSO only), and sensitivity was apparent for the positive control *mre11*Δ and *rad50*Δ strains that are known to be CPT-sensitive [25]. Interestingly, differential sensitivity was not observed for wild-type and *rad27*Δ strains or for the *rad27::polβ-D256A* and *rad27::polβ-3K* strains. Finally, similar results were obtained when the strains were exposed to ionizing radiation (Figure 7B), a DNA damaging agent, like CPT, that is not expected to result in repair intermediates with the 5′-dRP group. Together, these observations are consistent with the idea that MMS sensitivity observed in the *rad27Δ* and *rad27::polβ-3K* strains was due to the persistence of the 5′-dRP group.

## Discussion

*S. cerevisiae* cells lacking Rad27 exhibited hypersensitivity to MMS, and we were able to examine the reason for this cytotoxicity by expression of human Pol β. By removing the *RAD27* ORF and replacing it with the *POLβ* ORF, we found that wild-type Pol β expression was sufficient to partially rescue the MMS hypersensitivity conferred by the *RAD27* deficiency. This rescue was provided by the 5′-dRP lyase function of Pol β rather than the DNA polymerase gap-filling function. Thus, the separation of function for the two Pol β enzyme activities provided a useful tool toward attributing the cytotoxicity to persistent 5′-dRP groups, as opposed to persistent unfilled gaps or multinucleotide strand-displacement flaps.

### Importance of removing the 5′-dRP group

As discussed above, the necessity of the Pol β lyase domain for MMS sensitivity rescue indicates that the primary protective function against MMS for human Pol β in budding yeast was removal of cytotoxic 5′-dRP groups. A similar requirement for 5′-dRP group removal is also observed for MMS hypersensitivity in mammalian cells [26]. The fact that both of these eukaryotic organisms require this activity underscores the importance for further investigation into the cytotoxic effects of the blocked 5′-end. A need for understanding the various roles of the 5′-dRP group has been highlighted in studies of BER in mammalian cells. Here, poly (ADP-ribose) polymerase (PARP) inhibitor sensitization of cells to MMS-induced killing is dependent on the presence of the 5′-dRP group [27]. The topic is timely as PARP inhibitors are currently being evaluated in cancer chemotherapy [28]. Although the budding yeast system does not contain the PARP enzyme, this system can nonetheless informs us about the metabolism of the 5′-dRP group in BER intermediates.

One likely outcome of failure to remove the 5′-dRP group is a block in DNA ligation, creating persistent strand breaks. Previous reports demonstrated that MMS treatment in strains lacking Rad27 produced single-strand breaks [16], [29], but did not address the removal of the 5′-dRP group specifically. Our results suggested a key function of Rad27 in protection against MMS is to unmask a ligatable strand with a 5′-dRP blocked end, but this effect may be to enable a repair system other than BER. Interestingly, the wild-type extract showed minimal *in vitro* BER activity (Figures 3 and 4). Thus, any short gaps formed after Rad27 excision did not appear to be substrates for the endogenous DNA polymerases. Yet, removal of the 5′-dRP group also could be a critical step in multiple other types of DNA repair, where 5′-dRP removal is necessary for efficient ligation. The fact that our genetic background was lacking only in Rad27, and not other known DNA end-tailoring enzymes, suggests these enzymes were unable to process substrates with the 5′-dRP group. It is also noteworthy that yeast Trf4, known to possess 5′-dRP lyase activity [30], appeared unable to reverse the MMS sensitivity. Nevertheless, endogenous Trf4 activity may have provided for the weak *in vitro* BER product formation observed with extract from the *rad27*::*polB*-*3K* strain (Figures 3 and 4). In wild-type cells, it seems likely that Rad27 contributes the major 5′-dRP removal function through flap excision.

We chose not to delete *POL4*, the X-family polymerase in *S. cerevisiae* [24], [31], as it has not been implicated in gap filling during BER, but instead is involved in non-homologous end-joining [32], [33]. The observation of a lack of *in vitro* BER (Figures 3 and 4) in the wild-type extract was not surprising in light of earlier results [24] and suggests the gap-filling functions of yeast Pol 4 have evolved to specialize in the non-homologous end-joining branch of DNA repair. Since we found that the polymerase function of Pol β was dispensable for MMS rescue, it is likely that yeast utilizes Rad27 to remove the cytotoxic 5′-dRP group and that another repair pathway or process is recruited to complete the repair.

## Supporting Information

Figure S1 **Evidence of pol** β**-mediated BER in** ***S. cerevisiae*** **strain carrying human pol** β. (A) Schematic representations of the substrate and the reaction scheme are shown. (B) *In vitro* BER capacity of *S. cerevisiae* extracts. Repair reactions were incubated either with extracts from *wild-type* (lanes 1–3), *rad27::POLβ* (lanes 4–6), or *rad27::POLβ* supplemented with human DNA ligase I (lanes 7–9), respectively. Reaction mixtures (15 µl each) were assembled on ice as described under Material and Methods. The repair was initiated by transferring the reaction mixtures to 35°C. Aliquots (4.5 µl each) were withdrawn at 10, 20 and 40 min. The repair reaction was terminated by addition of an equal volume of DNA gel-loading buffer. After incubation at 75°C for 2 min, the reaction products were separated by electrophoresis in a 16% polyacrylamide gel containing 8 M urea. A Typhoon PhosphorImager was used for gel scanning and imaging. The positions of ligated BER product and un-ligated BER intermediate are indicated.
(TIF)
