# Supplementary figures and images for: Evidence for Abasic Site Sugar Phosphate-Mediated Cytotoxicity in Alkylating Agent Treated Saccharomyces cerevisiae
Source: PLoS One. 2012 Oct 29;7(10):e47945. doi: 10.1371/journal.pone.0047945 (PMC3483300; doi:10.1371/journal.pone.0047945)

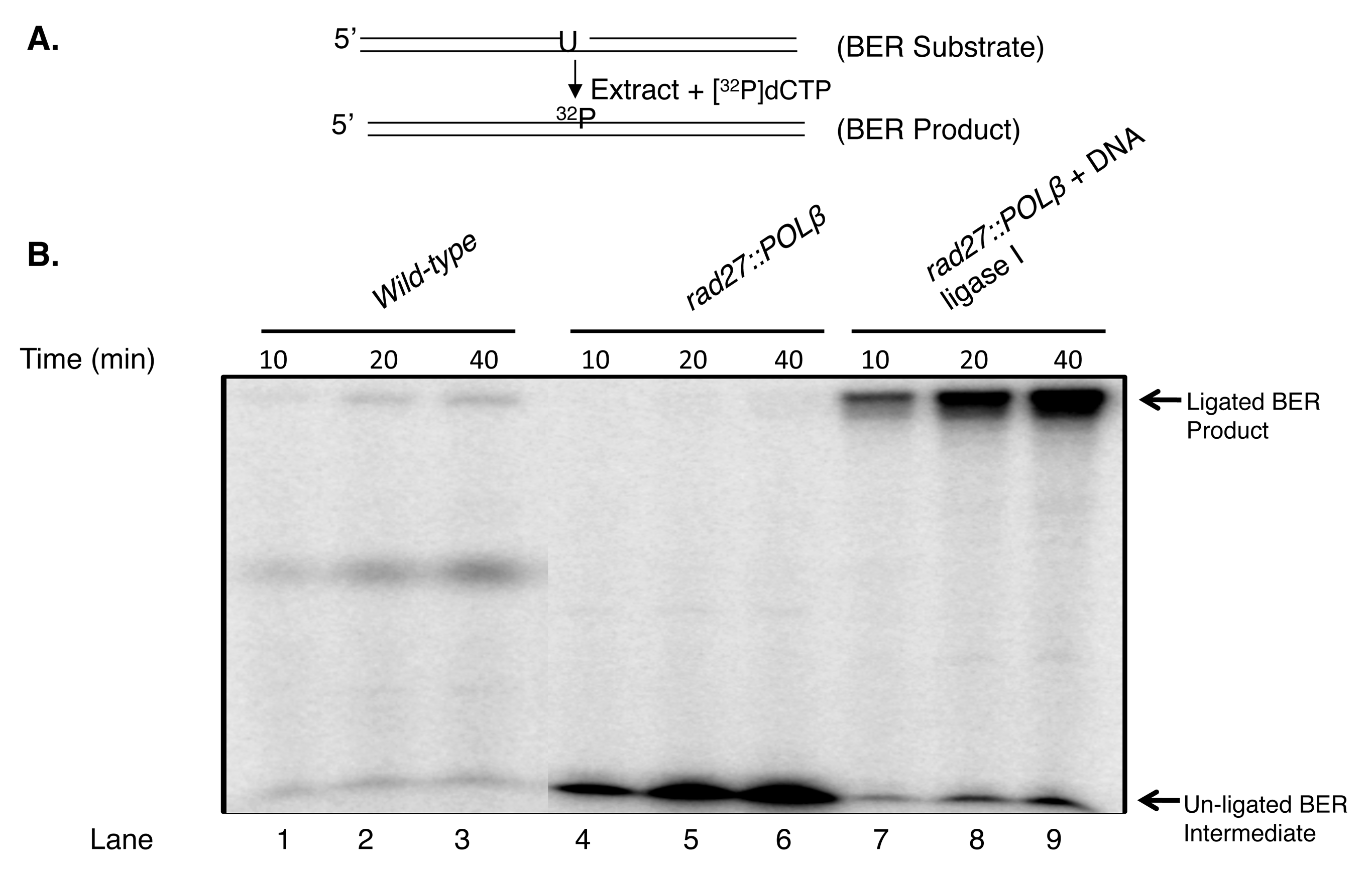

Supplement: Figure S1 — Evidence of pol β-mediated BER in S. cerevisiae strain carrying human pol β. (A) Schematic representations of the substrate and the reaction scheme are shown. (B) In vitro BER capacity of S. cerevisiae extracts. Repair reactions were incubated either with extracts from wild-type (lanes 1–3), rad27::POLβ (lanes 4–6), or rad27::POLβ supplemented with human DNA ligase I (lanes 7–9), respectively. Reaction mixtures (15 µl each) were assembled on ice as described under Material and Methods. The repair was initiated by transferring the reaction mixtures to 35°C. Aliquots (4.5 µl each) were withdrawn at 10, 20 and 40 min. The repair reaction was terminated by addition of an equal volume of DNA gel-loading buffer. After incubation at 75°C for 2 min, the reaction products were separated by electrophoresis in a 16% polyacrylamide gel containing 8 M urea. A Typhoon PhosphorImager was used for gel scanning and imaging. The positions of ligated BER product and un-ligated BER intermediate are indicated. (TIF) [file pone.0047945.s001.tif]
